# Permissive hypercapnia in acute respiratory distress syndrome: physiological mechanisms and clinical implications

**DOI:** 10.4103/mgr.MEDGASRES-D-25-00113

**Published:** 2026-01-06

**Authors:** Giacoma Teri, Patricia RM Rocco, Sergio Lassola, Denise Battaglini

**Affiliations:** Department of Surgical Sciences and Integrated Diagnostics (DISC), University of Genoa, Genoa, Italy; Anesthesia and Intensive Care, IRCCS Ospedale Policlinico San Martino, Genoa, Italy; Laboratory of Pulmonary Investigation, Carlos Chagas Filho Institute of Biophysics, Federal University of Rio de Janeiro, Rio de Janeiro, Brazil; Department of Anesthesia and Intensive Care, Santa Chiara Hospital, Trento, Italy

Clinically, acute respiratory distress syndrome (ARDS) is characterized by arterial hypoxemia, bilateral infiltrates on lung imaging, and non-cardiogenic pulmonary edema resulting from increased alveolar-capillary permeability. This is accompanied by increased intrapulmonary shunt, elevated alveolar dead space, and decreased respiratory system compliance. The Berlin definition provides a standardized framework for diagnosing ARDS; however, it may not fully encompass the clinical heterogeneity of the syndrome, potentially limiting diagnostic sensitivity and specificity. Emerging evidence supports broadening diagnostic criteria to incorporate non-invasive modalities such as high-flow nasal oxygen, pulse oximetry as a surrogate for arterial blood gases, and lung ultrasound—particularly relevant in resource-limited settings where conventional diagnostics may be unavailable.^1^,^2^

Despite advances in the diagnosis and supportive management of ARDS, mortality remains unacceptably high, ranging from 30% to 45%. The primary causes of death are sepsis and multiorgan failure, rather than refractory hypoxemia alone. A major factor contributing to the persistently high mortality associated with ARDS is the lack of effective, disease-specific therapies. Current management remains largely supportive, with lung-protective ventilation (LPV) as its cornerstone. The primary objectives of LPV are to minimize ventilator-induced lung injury (VILI), ensure adequate oxygenation, and reduce oxygen consumption and patient respiratory effort. Recent guidelines from the European Society of Intensive Care Medicine (2023)^1^ and American Thoracic Society (2024)^2^ recommend a tidal volume of 6–8 mL/kg of ideal body weight, maintaining plateau pressures below 30 cmH_2_O, and using individualized positive end-expiratory pressure (PEEP) titration to balance alveolar recruitment and the risk of overdistension. However, LPV is not without limitations. Reducing tidal volume may lead to carbon dioxide (CO_2_) retention and permissive hypercapnia. While hypercapnia has been proposed to confer protective effects against VILI, emerging evidence suggests that it may have deleterious consequences in certain ARDS subphenotypes,^3^ highlighting the need for personalized approaches.

This perspective explores the physiological rationale and evolving clinical implications of permissive hypercapnia in the management of ARDS, with particular attention to its therapeutic potential and possible adverse effects in contemporary critical care.

**Understanding carbon dioxide and its physiological role:** Carbon dioxide, produced during cellular metabolism, is crucial for several bodily functions. It helps cellular respiration, influences how oxygen binds to hemoglobin, and maintains acid–base balance. Additionally, CO_2_ exerts significant effects on both vascular tone and immune function. On average, our body produces 15,000–20,000 millimoles of CO_2_ each day, primarily through the complete oxidation of carbohydrates and lipids.

Once produced, CO_2_ is transported from peripheral tissues to the lungs via the bloodstream and eliminated through alveolar gas exchange. This transmembrane diffusion is governed by Fick’s Law, which states that the rate of gas transfer (Gv) is proportional to the surface area available for diffusion (A), the diffusion coefficient (D), and the partial pressure gradient (P_1_ − P_2_), and inversely proportional to the thickness of the alveolar–capillary membrane (T), expressed as:

Gv = A × D × (P_1_ − P_2_)/T

Under normal conditions, the diffusion capacity of CO_2_ is approximately 25 mL/min/mmHg. While this may increase during exercise, it can decline markedly in diseases such as emphysema or pulmonary fibrosis, where structural alterations impair gas exchange efficiency.

Arterial partial pressure of carbon dioxide (PaCO_2_) reflects systemic CO_2_ levels and is a principal regulator of arterial pH (pHa), as described by the Henderson–Hasselbalch equation:

pHa = 6.1 + log ([HCO_3_^−^] / (0.03 × PaCO_2_))

Tight regulation of PaCO_2_ is maintained by chemoreflex mechanisms. Central chemoreceptors located on the ventral surface of the medulla oblongata detect pH changes in the cerebrospinal fluid and adjust minute ventilation in response to elevations in PaCO_2_. This response is linear and continues until a new homeostatic setpoint is reached. Peripheral chemoreceptors—located in the carotid and aortic bodies—supplement this control by sensing reduced arterial oxygen tensión as well as changes in pH, and PaCO_2_.

In ARDS, the normal coupling between alveolar ventilation (V’) and pulmonary perfusion (Q’) is severely disrupted, resulting in profound ventilation–perfusion (V’/Q’) mismatch. Intrapulmonary shunt is the primary cause of hypoxemia, while increased alveolar dead space contributes significantly to hypercapnia. As ARDS progresses into the fibroproliferative phase, structural remodeling and altered pulmonary mechanics further exacerbate V’/Q’ mismatch, impeding effective CO_2_ elimination and oxygenation.

In this setting, LPV is recommended to mitigate further lung injury. However, the use of low V_T_, though essential for limiting VILI, can lead to CO_2_ retention, culminating in permissive hypercapnia.

**Permissive hypercapnia – concept and rationale:** Permissive hypercapnia was first introduced in the early 1990s following two seminal case series in which reduced tidal volumes (V_T_) and airway pressures — despite resulting in elevated PaCO_2_ — were associated with lower in-hospital mortality than predicted by APACHE II scores (Acute Physiologic Assessment and Chronic Health Evaluation).^4^ This marked a paradigm shift in ventilatory management and laid the groundwork for subsequent large-scale randomized controlled trials in ARDS patients, which confirmed that LPV strategies reduce VILI, improve weaning and improve patient survival.^5^ Therefore, permissive hypercapnia is now an accepted and often necessary component of ARDS management. Hypercapnia exerts diverse physiological effects that may offer protective benefits in the context of ARDS:

***Pulmonary effects:*** Elevated CO_2_ increases lung compliance by enhancing alveolar surfactant production and function in acidic environments, while also leading to hypoxic pulmonary vasoconstriction through changes in membrane potential. Together, these factors enhance the V’/Q’ mismatch. Hypercapnia also reduces alveolar−capillary permeability by improving pulmonary edema, inhibits hypoxia-induced chronic pulmonary hypertension, and provides protection against neonatal chronic lung injury.

***Cardiovascular effects:*** Although hypercapnia can decrease vascular smooth muscle and myocardial contractility in isolated hearts, it also stimulates the sympathetic system, thus raising heart rate, decreasing afterload, increasing preload, and ultimately cardiac output.

***Microvascular and oxygenation effects:*** Hypercapnia shifts the oxyhemoglobin dissociation curve to the right facilitating O_2_ release (Bohr effect) and promotes microvascular vasodilation improving oxygen supply at both arterial and tissue levels.

***Anti-inflammatory and cytoprotective effects:*** CO_2_ increases the production of nitric oxide, which inhibits the activity of xanthine oxidase and the production of superoxide radicals by neutrophils. Hypercapnia also attenuates inflammation by inhibiting nuclear factor kappa B (NF-κB).^6^ Lastly, by reducing intracellular calcium excess, CO_2_ reduces ischemia-reperfusion cellular damage. These properties suggest that by enhancing arterial and tissue oxygenation, lowering mechanical stress, and modifying inflammatory responses, permissive hypercapnia may aid in slowing the development and intensity of ARDS.

Despite these potential benefits, permissive hypercapnia is not without risks. Of particular concern are its hemodynamic, neurologic, respiratory muscle, and acid–base effects:

***Hemodynamic effects:*** Hypercapnia-induced pulmonary vasoconstriction can aggravate or precipitate pulmonary hypertension and right ventricular dysfunction, potentially decreasing cardiac output and renal perfusion. This may contribute to acute kidney injury through both low forward flow and venous congestion.

***Neurological effects:*** CO_2_ increases cerebral blood flow by 1–2 mL/100 g/min for every 1 mmHg increase in PaCO_2_, which can lead to increased intracranial pressure. Hypercapnia also produces increased sympathetic and decreased parasympathetic tones by increasing brain levels of glutamine and gamma-aminobutyric acid, and decreasing levels of glutamate and aspartate, resulting in depressed minute volume and work of breathing in inspiration. Given the narrow safety ranges of CO_2_ for cerebral protection, the management of patients with acute brain injury is particularly challenging.

**Respiratory muscle dysfunction:** Hypercapnia impairs diaphragmatic function in spontaneously breathing patients through alterations in electrical signaling of the phrenic nerve afferent pathways and represses skeletal muscle ribosomal gene expression through downregulation of 45S preRNA, with consequent dysfunctional protein anabolism.

**Acid–base imbalance:** As dictated by the Henderson–Hasselbalch equation, rising PaCO_2_ results in respiratory acidosis. Maintaining pH within physiological limits is essential for enzymatic activity, cellular integrity, and survival.

**Figure 1:**
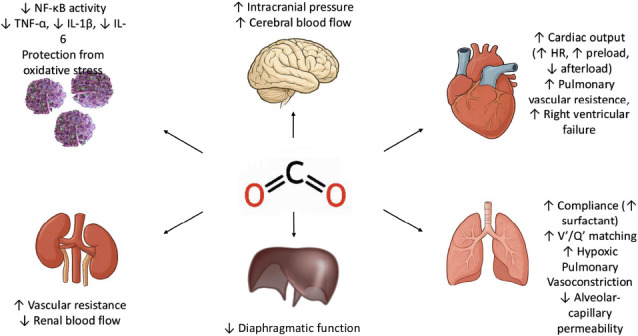
Systemic effects of permissive hypercapnia. Permissive hypercapnia exerts wide-ranging physiological effects across multiple organ systems. In the lungs, it improves ventilation–perfusion (V′/Q′) matching and promotes alveolar stability by enhancing surfactant production and optimizing surface tension properties under acidic conditions. Cardiovascularly, hypercapnia can increase cardiac output through mechanisms such as elevated preload, reduced afterload, and augmented sympathoadrenal activity. At the vascular and immunological levels, it exerts anti-inflammatory and cytoprotective effects by downregulating proinflammatory cytokines — including TNF-α, IL-1β, and IL-6 — and by inhibiting oxidative stress pathways mediated by NF-κB. Despite these potential benefits, hypercapnia may also carry significant risks. Within the central nervous system, CO_2_-induced vasodilation can increase cerebral blood flow and intracranial pressure, complicating management in patients with neurological injury. In the kidneys, hypercapnia has been linked to increased renal vascular resistance and diminished renal perfusion, potentially contributing to acute kidney injury. These systemic effects underscore the critical need for continuous monitoring and individualized adjustment of ventilatory strategies to balance therapeutic advantages against physiological risks. Created with Microsoft PowerPoint. HR: Heart rate; IL-1β: interleukin-1 beta; IL-6: interleukin-6; NF-κB: nuclear factor kappa B; TNF-α: tumor necrosis factor alpha; V′/Q′: ventilation–perfusion.

**Figure 1** illustrates the multifaceted physiological effects of permissive hypercapnia in ARDS.

**Management of hypercapnia in ARDS:** Several strategies can be employed to mitigate the adverse effects of hypercapnia while preserving the benefits of LPV:

***Buffering respiratory acidosis:*** Tris-hydroxymethyl aminomethane has shown efficacy in correcting acidosis and reducing PaCO_2_ levels, thereby improving hemodynamic stability and myocardial function. In contrast, sodium bicarbonate relies on adequate CO_2_ clearance; in conditions with impaired ventilation such as ARDS, its use may exacerbate CO_2_ accumulation and worsen hypercapnia.

***PEEP titration and recruitment maneuvers:*** Personalized PEEP titration can decrease physiologic dead space and promote more homogeneous ventilation. However, excessive PEEP carries risks of barotrauma and hemodynamic compromise. Recruitment maneuvers, which involve transient increases in airway pressure to reopen collapsed alveoli, may improve gas exchange but pose risks of overdistension and hemodynamic instability. Consequently, current guidelines do not recommend their routine use.^1^,^2^

***Prone positioning:*** Prone ventilation enhances oxygenation, reduces dead space, and improves ventilation distribution by decreasing lung stress and strain. It has been shown to reduce mortality in patients with moderate-to-severe ARDS and may aid in mitigating hypercapnia, particularly in responders.^1^

***Extracorporeal therapies:*** For patients with refractory hypercapnia, extracorporeal modalities such as venovenous extracorporeal membrane oxygenation or extracorporeal CO_2_ removal (ECCO_2_R) can be considered. Venovenous extracorporeal membrane oxygenation provides full gas exchange support and is recommended for selected cases of severe ARDS, although its availability and eligibility criteria vary widely. ECCO_2_R allows ultraprotective ventilation by enabling further reductions in tidal volume and respiratory rate. Recent studies have demonstrated that ECCO_2_R reduces PaCO_2_, increases pH, and lowers driving pressure within the first 48 hours of therapy.^7^ However, no definitive mortality benefit has been shown to date, and its routine use outside clinical trials is not currently advised.^1^

**Clinical outcomes and ongoing controversy:** The impact of permissive hypercapnia on clinical outcomes remains a subject of debate. In a secondary analysis of the LUNG SAFE study, hypercapnia was not associated with clinical outcomes, whereas hypocapnia in the first two days of mechanical ventilation was linked to increased Intensive Care Unit mortality in patients with mild to moderate ARDS.^8^ Similarly, a multicenter observational study found no clear benefit or harm from hypercapnia.^9^ In contrast, other studies have reported adverse outcomes, indicating a link between severe hypercapnia and higher intensive care unit mortality in ARDS patients receiving LPV.^10^ A recent systematic review and meta-analysis sought to resolve these conflicting findings by distinguishing permissive hypercapnia, resulting from LPV, from imposed hypercapnia, defined as hypercapnia already present a priori regardless of the ventilation strategies used. Imposed hypercapnia may reflect disease severity rather than serve as a therapeutic target, as studies have shown that permissive hypercapnia resulting from lung-protective strategies is associated with improved outcomes, whereas deliberately targeting elevated PaCO_2_ levels has been linked to worse prognoses.^3^

**Conclusions:** Permissive hypercapnia is a fundamental component of modern LPV supporting strategies in the management of ARDS. By enhancing arterial and tissue oxygenation, attenuating pulmonary and systemic inflammation, and improving V’/Q’ matching, permissive hypercapnia may physiologically optimize gas exchange and reduce ventilator-induced lung injury. However, its application carries important risks, including hemodynamic compromise and acid–base disturbances, which must be carefully monitored and balanced. The overall clinical impact of permissive hypercapnia remains under active investigation. While observational data have produced mixed results, emerging evidence suggests that patient outcomes may depend on the context in which hypercapnia develops. When hypercapnia arises because of well-implemented LPV, it may confer physiological benefits and improve survival. In contrast, hypercapnia that develops independently of LPV — referred to as imposed or unintentional hypercapnia — may reflect more severe underlying lung pathology and has been associated with worse outcomes.

Further mechanistic investigations and well-designed prospective randomized clinical trials are essential to more precisely define the physiological thresholds, patient subphenotypes, and clinical contexts that modulate the balance of risks and benefits associated with permissive hypercapnia. This evidence will be critical to inform tailored treatment strategies, enabling personalized ventilation approaches that optimize outcomes in patients with ARDS.
